# Intravenous Drug Use and Hepatitis C Virus in Iran

**DOI:** 10.5812/kowsar.1735143X.797

**Published:** 2012-01-20

**Authors:** Mehdi Zobeiri, Peyman Adibi, Seyed Moayed Alavian

**Affiliations:** 1 Department of Internal Medicine, Kermanshah University of Medical Sciences, School of Medicine, Imam Reza Hospital, Kermanshah, IR Iran; 2 Department of Gastroenterology, Isfahan University of Medical Sciences, Isfahan, IR Iran; 3 Baqiyatallah Research Center for Gastroenterology and Liver Diseases, Baqiyatallah University of Medical Sciences, Tehran, IR Iran

**Keywords:** Substance Abuse, Intravenous, Hepatitis C

HCV infection is a global public health problem and can progress to cirrhosis, hepatic decompensation, hepatocellular carcinoma, and death, with asymptomatic conditions and few short-term effects in the beginning [1][2]. It is expected that over the next decade, despite a declining incidence of new infections, mortality and associated costs will increase [1]. Worldwide prevalence rates range from 0.01 to 20%, and the World Health Organization (WHO) estimates that up to 3% of the world's population (170 million) have been infected with HCV [3]. Worldwide hepatitis C data show significant prevalence rates in high-risk populations, ranging from 30% to 50%, with injecting drug use being the predominant risk factor [4][5]. In most cases, HCV is spread parenterally, and research has identified intravenous and intranasal drug use, exposure to infected blood and blood products, and high-risk behaviors as risk factors in parenteral transmission [1]. The variation in HCV prevalence creates the need for different preventive methods, community interventions, and therapeutic strategies based on economic and social variance [6]. Drug addicts are a high-risk group for acquiring one or more parenterally transmitted infection, such as HIV, HCV, HBV and HTLV-1 [7].

In Iran opiates are the most commonly abused drug type, and it has been estimated that 2.8% of adults ages 15 to 64 are opiate abusers [8][9]. Also, data from the 2006 Spengler Forum Index revealed that 12.2% (180,000) of drug abusers were injecting drug users (IDUs; [10]). Another study that year with a different dataset estimated that there were 200 to 300 thousand IDUs with a mean age of 33 ± 8.9 years in Iran [11]. Nearly 40% of Iranian prisoners were IDUs, and the prevalence of HCV infection among this population has been reported to be between 38 and 90% [12][13]. HCV seroprevalence among prison inmates varies markedly from country to country, but as a whole, research has shown that drug-abusing prisoners and habitual injectors were 90 times more likely than healthy blood donors and the general population to be infected with HCV [14][15][16]. The risk of testing positive for HCV among prisoners was found to be associated with marital status, duration of drug use, IV drug use, shared injection equipment, duration of incarceration, tattoos, and number of times in prison [13]. The point prevalence of sterile-syringe use was 85% among IDUs, but a history of sharing injection equipment was reported between 50 and 70% of them [9][17]. Another study has shown that tattoos might have a higher risk of HCV infection when given in prison [18]. Mirahmadizadeh et al. found that the proportions of unsafe and unprotected homo- and heterosexual contact among IDUs were 19.4% and 37.4 %, respectively [19].

Today, injecting drug abuse is a major and perhaps the most important risk factor for HCV infection [20]. Intravenous drug abusers not only have the highest prevalence of HCV infection but also constitute a potential reservoir of HCV in the community [6]. Injecting drug use and health care provision are the most important risk factors for HCV infection, and the number of HCV-infected persons continues to grow [21]. In early reports from Tehran in 2001 by Zali et al., ELISA tests were HCV Ab positive for 45% of imprisoned IDUs [22]. About 38% of IDU prisoners in the western province of Hamadan, 47% in the northwestern province of Zanjan, 52% in Tehran, 64.8% in the southern cities of Bandar Abbas and Roodan, and 88.9% in the northern of Guilan were HCV Ab positive, whereas the rate in non-IDUs in prison was much lower [1][13][23][24]. Iran has one of the highest per capita numbers of opioid users in the world, with a substantial and potentially growing proportion of IDUs [8][9]. The dramatic growth in the number of prisoners involved in high-risk behaviors and the high rates of community reentry emphasize the need for screening, detection, and treatment of HCV infection as well as harm-reduction programs to help curtail the rise of HCV infection in incarcerated populations and the general population [1]. Because many drug users spend time in prison, it is an appropriate to break the cycle of infection by multidisciplinary interventional approaches targeted at controlling the further spread of these infections among prisoners and to the general population. Intervention efforts have expanded progressively to include comprehensive HIV prevention with epidemiological surveillance, educational programs, research activities, and methadone-maintenance therapy, but they must further incorporate components such as HCV-specific educational programs and encourage inmates to avoid tattooing while in prison [25].
